# Antimicrobial and anti-Quorum Sensing activities of selected medicinal plants of Ethiopia: Implication for development of potent antimicrobial agents

**DOI:** 10.1186/s12866-016-0765-9

**Published:** 2016-07-11

**Authors:** Ketema Bacha, Yinebeb Tariku, Fisseha Gebreyesus, Shibru Zerihun, Ali Mohammed, Nancy Weiland-Bräuer, Ruth A. Schmitz, Mulugeta Mulat

**Affiliations:** Depatment of Biology, College of Natural Sciences, Jimma University, Jimma, Ethiopia; Department of Chemistry, College of Natural Sciences, Jimma University, Jimma, Ethiopia; Department of Horticulture, College of Agriculture, Adigrat University, Adigrat, Ethiopia; Department of Horticulture and Plant Science, College of Agriculture and Natural Resources Management, Gambella University, Gambella, Ethiopia; Departemnt of Postharvest Management, College of Agriculture and Veterinary Medicine, Jimma University, Jimma, Ethiopia; Institut für Allgemeine Mikrobiologie, Christian-Albrechts-Universität zu Kiel, Kiel, Germany; Derpartment of Biology, College of Natural and Computational Sciences, Wollo University, Dessie, Ethiopia

**Keywords:** Alternative medicine, Drug resistance, Ethiopia, Medicinal plants, MIC, Quorum Sensing, Quorum Quenching

## Abstract

**Background:**

Traditional medicinal plants have been used as an alternative medicine in many parts of the world, including Ethiopia. There are many documented scientific reports on antimicrobial activities of the same. To our knowledge, however, there is no report on the anti-Quorum Sensing (Quorum Quenching, QQ) potential of traditional Ethiopian medicinal plants. As many of the opportunistic pathogenic bacteria depend on Quorum Sensing (QS) systems to coordinate their virulence expression, interference with QS could be a novel approach to control bacterial infections. Thus, the aim of this study was to evaluate selected medicinal plants from Ethiopia for their antimicrobial activities against bacterial and fungal pathogens; and to assess the interference of these plant extracts with QS of bacteria.

**Methods:**

Antimicrobial activities of plant extracts (oil, resins and crude extracts) were evaluated following standard agar diffusion technique. The minimum inhibitory concentrations (MIC) of potent extracts were determined using 96 well micro-titer plates and optical densities were measured using an ELISA Microplate reader. Interference with Quorum Sensing activities of extracts was determined using the recently established *E. coli* based reporter strain AI1-QQ.1 and signaling molecule *N*-(ß-ketocaproyl)-L-homoserine lactone (3-oxo-C6-HSL).

**Results:**

Petroleum ether extract of seed of *Nigella sativa* exhibited the highest activity against both the laboratory isolated *Bacillus cereus* [inhibition zone (IZ), 44 ± 0.31 mm] and *B. cereus* ATCC 10987 (IZ, 40 ± 2.33 mm). Similarly, oil extract from mature ripe fruit husk of *Aframomum corrorima* and mature unripe fruit of *A. corrorima* revealed promising activities against *Candida albicans* ATCC 90028 (IZ, 35 ± 1.52 mm) and *Staphylococcus aureus* DSM 346 (IZ, 25 ± 1.32 mm), respectively. Antimicrobial activities of oil extract from husk of *A. corrorima* and petroleum ether extract of seed of *N. sativa* were significantly higher than that of the control antibiotic [Gentamycin sulfate, (IZ, 25–30 mm)]. The lowest MIC value (12.5 mg/mL) was recorded for oil from husk of *A. corrorima* against *Pseudomonas aeruginosa*. Of the total eighteen extracts evaluated, two of the extracts [Methanol extract of root of *Albiza schimperiana* (ASRM) and petroleum ether extract of seed of *Justica schimperiana* (JSSP)] interfered with cell-cell communication most likely by interacting with the signaling molecules.

**Conclusion:**

Traditional medicinal plants from Ethiopia are potential source of alternative medicine for the local community and scientific research in search for alternative drugs to halt challenges associated with the emerging antimicrobial resistance. Furthermore, the Quorum Quenching activities observed in two of the plant extracts calls for more comprehensive evaluation of medicinal plants for the control of many bacterial processes and phenotypic behaviors such as pathogenicity, swarming, and biofilm formation. Being the first assessment of its kind on the potential application of Ethiopian traditional medicinal plants for interference in microbial cell-cell communication (anti-Quorum Sensing activities), the detailed chemistry of the active compounds and possible mechanism(s) of actions of the bio-molecules responsible for the observed interference were not addressed in the current study. Thus, further evaluation for the nature of those active compounds (bio-molecules) and detailed mechanism(s) of their interaction with microbial processes are recommended.

## Background

Traditional medicinal plants have indefinite therapeutic value worldwide. The World Health Organization estimates that up to 80 % of the world population still relies on traditional remedies [1] with more than 35,000 plant species being used for medication purpose in various human cultures [2]. Ethiopia is the origin and center of biodiversity for many plant species and is one of the six plant biodiversity-rich countries of Africa, being home for about 6,500 species of higher plants, 12 % of which are endemic to the country [3, 4]. Due to its long history of practice and safety, traditional medicine has become an integral part of the Ethiopian culture. Accordingly, about 80 % of human population and 90 % of livestock rely on traditional medicine for treatment of diseases [5]. The emergence of antibiotic resistant microbial strains and increasing failure of the available chemotherapeutics made the search for microbiologically active medicinal plants a necessity [6].

In host parasite interaction, pathogenic bacteria generally deploy two major strategies: invading host tissues (invasiveness), and producing toxins (Toxigenesis) [7, 8]. Invasiveness encompasses mechanisms for colonization (adherence and initial multiplication), ability to bypass or overcome host defense mechanisms, and the production of extracellular substances which facilitate invasion [8]. In toxigenesis, however, bacteria produce the cell associated Endotoxins or Exotoxins. Plant extracts with potential antibacterial activity interfere with one or more of the above bacterial pathogenicity strategies.

In addition to inhibition of microbial growth, natural products might also play a significant role in interfering microbial cell-cell communication processes, commonly called Quorum Sensing (QS) [9]. QS allows the perception of population density by small signaling molecules, called auto-inducers, and modifies gene expression in response to the population density [10, 11]. It controls a wide spectrum of processes and phenotypic behaviors, including stress resistance, production of toxins and secondary metabolites, pathogenicity, swarming, and biofilm formation [11, 12]. Unlike humans and mammals that possess immune systems to defend against invaders, plants are lacking such sophisticated immunity to ward off invading pathogens. Thus, instead of relying on cellular and biochemical defense systems, plants may have evolved alternative defense mechanisms, including production of anti-QS compounds, which can be used to defeat QS pathogens [9]. According to González and Keshavan [13], eukaryotes have evolved efficiently to manipulate bacterial QS systems and protect themselves from attacks by pathogens. For instance, interference with QS systems were clearly observed among marine red alga [14], human keratinocytes [15], and human airway epithelial cells [16].

Although numerous studies have been undertaken, and have revealed the antimicrobial potential of traditional medicinal plants being used in various parts of Ethiopia [17–23], the effects of those plant extracts on bacterial cell-cell communication were not evaluated so far. Many bacteria are thought to utilize chemical signaling systems to control cellular behaviors in response to local bacterial population density for effective colonization and manipulation of host organisms, such as in disease and symbiosis [12]. Jamming such bacterial communication systems has, therefore, become an attractive target for intervention strategies to mitigate and treat infectious diseases. Gram-negative bacteria use acylated homoserine lactones as auto-inducers, while Gram-positive bacteria use oligopeptides to communicate. Report on quorum sensing and quorum sensing molecules from fungi are rare [24–26]. Two molecules have been described from the human commensal and pathogenic fungus *Candida albicans*, namely farnesol and tyrosol [24, 25]. The human opportunistic yeast, *Cryptococcus neoformans* was also reported to produce peptide type signaling molecules [26].

Irrespective of the inducer molecules used, if bacteria use the QS circuit for such diverse arrays of activities, identification of molecules that interfere with the inducer molecules or its expression could have paramount importance particularly in the control of microbial diseases. Thus, this study was designed to evaluate the antimicrobial activities of selected traditional medicinal plants widely used in Ethiopia, and additionally to assess effects of extracts of the plants on cell-cell signaling. The findings of the current study could pave the way for further detailed study on traditional medicinal plant resources of Ethiopia for wider application in food and pharmaceutical industries.

## Methods

### Plant collection

Traditional medicinal plants were collected from two districts of Jimma Zone (Kersa and Omo Nada) and also purchased from open markets of Serbo, Assendabo, and Jimma towns, Southwest Ethiopia. The collected traditional medicinal plants were labeled with their date of collection, location, medicinal uses and approximate dosages of administration based on the information gathered from the local healers (informants). The taxonomic categories of the plants were identified assisted by professional taxonomist at Jimma University; Department of Biology and Voucher specimens were deposited at Jimma University Herbarium.

### Preparation of plant extracts

A total of 18 plant extracts were prepared following standard methods of plant preparation for extraction as described earlier [27]. Briefly, the collected plant specimens were properly cleaned and air dried under shade on wire mesh bed for 15 days and finally powdered manually to suitable size with metal mortar and pestle. The finger rhizomes and main rhizomes of *Curcuma longa* were pre-cooked at 80 ^0^C for 75 min and at 90 ^0^C for 75 min, respectively, to facilitate the extraction process. The powdered plant materials were subsequently extracted successively with petroleum ether, chloroform, methanol and water (the details are as given below).

### Isolation of volatile oil

Volatile constituents of the study plants (*Curcuma longa, Aframomum corrorima* and *Nigella sativa*) were obtained by hydro-distillation [28] using 60–100 g samples on a glass type Clevenger apparatus for 3–4 h after the mixture started boiling. The crude oil was then separated and the remaining moisture content was absorbed by adding anhydrous sodium sulphate. The oil was placed in a glass vial sealed with parafilm and kept in a refrigerator (2–4 °C) protected from direct light.

### Isolation of Oleo resins and crude extracts

Oleo resins and crude extracts of the study plants (*Curcuma long, Aframomum corrorima, Albiza schimperiana, Justica schimperiana, Erythrinia brucei, Vernonia amygdalina, Nigella sativa and Ocimum sauve)* were obtained by Soxhlet extraction [28] of 100 g samples in 1.5 L of the required solvents (petroleum ether, methanol, or chloroform) for 6 h. Residual solvents were evaporated using a rotary evaporator at 40 °C. The concentrated extracts were placed in glass vials, sealed with parafilm and kept in a refrigerator (2–4 °C) protected from direct light.

### Culture media and test strains

Muller Hinton agar (MHA) and Potato Dextrose agar (PDA) were used for antimicrobial activity tests, while LB (Luria Bertani) top agar was used for Quorum Quenching assay. A total of five bacterial strains [*Esherichia coli* K12, DSM 498*; Pseudomonas aeruginosa,* DSM 1117*; Staphylococcus aureus,* DSM 346*; Bacillus cereus* ATCC 10987; *B. cereus* (lab isolate)] and *Candida albicans* ATCC 90028 were used to evaluate the antimicrobial activities of plant extracts. Besides representing bacteria of Gram positive and Gram negative categories, these bacterial strains are among the ecologically rich (*Pseudomonas aeruginosa, Bacillus*), hence potential contaminants, and common inhabitants of human body including skin (*S. aureus*) and Gastro Intestinal Tract (*E. coli*). *Candida albicans* are opportunistic fungal pathogens of public health importance especially in immune-compromised individuals. Initiation of pathogenicity processes in some of these bacterial strains (including enterotoxin production in *S. aureus*) relies on density dependent cell-cell signaling (QS) molecule, which in turn is the potential target of plant extracts. Thus, inclusion of the above bacterial and fungal strains is rational and justifiable.

### Determination of antimicrobial activities

Agar diffusion method was used to evaluate antimicrobial activities of the extracts according to Tambekar *et al* [29]. Briefly, 100 μl volume of an overnight culture of the reference strains were separately spread over the agar plates (MHA for bacterial strains and PDA for *Candida albicans*). Sterilized standard paper discs (6 mm diameter) were placed on the already inoculated culture plates and flooded with 10 μL volume of the extracts (aqueous, chloroform, petroleum ether, and methanol; conc. 500 mg/mL). Gentamycin sulfate (conc. 1 μg/mL) and Nystatin (conc. 1 μg/mL) were used as positive controls for bacterial cultures and *Candida albicans,* respectively*.* Antibacterial/antifungal activities of the plant extracts against the test strains were determined after incubation of the test plates for 24 h at 37 °C (bacterial strains) or 48 h at room temperature (for *Candida albicans*) by measuring the diameter of zone of growth inhibition (IZ). The antimicrobial activity screening was conducted in triplicate experiments and results were an average (mean ± SD) values. A total of 18 plant extracts were analyzed (Table 1).

**Table 1:** Summary of traditional medicinal plants and their extracts evaluated for antimicrobial and Quorum Quenching (anti-Quorum Sensing) activities

Sample No	Code	Scientific name of plant with brief description	Type of Extract
1	ACFA1	*Aframomum corrorima* mature semi-ripe fruit acetone extract	Oleo resin
2	ACFA2	*Aframomum corrorima* mature unripe fruit acetone extract	Oleo resin
3	ACFA3	*Aframomum corrorima* mature ripe fruit acetone extract	Oleo resin
4	ACFO	*Aframomum corrorima* mature unripe fruit oil	Essential oil
5	ACHO	*Aframomum corrorima* mature ripe fruit husk oil	Essential oil
6	ASRM	*Albiza schimperiana* root methanol extract	Crude extract
7	CLRA1	*Curcuma longa* finger rhizome acetone extract	Oleo resin
8	CLRO1	*Curcuma longa* finger rhizome oil	Essential oil
9	CLRO2	*Curcuma longa* main rhizome oil	Essential oil
10	CLRA2	*Curcuma longa* main rhizome acetone extract	Oleo resin
11	EBBP	*Erythrinia brucei* stem bark petroleum ether extract	Crude extract
12	JSSP	*Justica schimperiana* seed petroleum ether extract	Crude extract
13	NSSO	*Nigella sativa* seed oil	Essential oil
14	NSSP	*Nigella sativa* seed petroleum ether extract	Crude extract
15	OSLC	*Ocimum sauve* leaf chloroform extract	Crude extract
16	VALC	*Vernonia amygdalina* leaf chloroform extract	Crude extract
17	VALM	*Vernonia amygdalina* leaf methanol extract	Crude extract
18	VALP	*Vernonia amygdalina* leaf petroleum ether extract	Crude extract

### MIC determination

The Minimum Inhibitor Concentration (MIC) was determined for those extracts that displayed potent antimicrobial activity against the test strains (activity guided). The standard 96 well micro-titer plate method as described earlier [30] was used with minor modifications to determine the lowest concentration of the extract that inhibit growth of the test strains (the presence or absence of growth was determined through measurement of difference in optical density before and after incubation instead of relying on color change). Accordingly, the initial stock concentration of the extract (25 mg/mL) was serially diluted in steps of 1:2 dilutions (25, 12.5, 6.25, 3.12, 1.5, 0.5, 0.25, 0.12 mg/mL) by transferring 100 μL of the stock extract into wells of micro-titer plate loaded with 100 μl nutrient broth, discarding the last 100 μl in the dilution series. Overnight cultures of the test strains (100 μl) were separately inoculated into the serially diluted extracts except for the culture free control, in a total volume of 200 μl. Absorbance was measured (at 530 nm and 340 nm for bacterial strains and *C. albicans,* respectively) before incubation for 24–48 h at 30–32 °C (for bacteria) and 48 h (for *C. albicans*) using ELISA Microplate Reader (SpectraMax Plus 384 Microplate Reader, Molecular Devices, USA). Absorbance was re-measured at the end of incubation to determine the final absorbance and compared with the initial measurement. Non-inoculated wells (wells loaded only with nutrient broth and DMSO, the solvent used to dissolve extracts) were used as negative control while wells with Gentamycin sulfate (conc. 1 μg/mL) and Nystatin (conc.1 μg/mL) were considered as positive controls. The MIC assays were carried out in duplicates.

### Quorum quenching assay

The recently established *E. coli* reporter strain AI1-QQ.1 was used to detect novel bio-molecules interfering with acyl homoserine lactone (AHL) based bacterial cell-cell communication in the plant extracts [11]. The *E. coli* reporter comprises a gene encoding a lethal protein fused to promoter induced in the presence of Quorum Sensing signal molecule AHL. Consequently, the *E. coli* strain is unable to grow in the presence of AHL signal molecules; unless a non-toxic QS-interfering compound is present. Accordingly, Quorum Quenching (QQ) screening plates were prepared as follows: LB top agar containing 0.8 % agar at 50 °C was supplemented with final concentrations of 100 μM *N*-(ß-ketocaproyl)-L-homoserine lactone (3-oxo-C6-HSL) (Sigma-Aldrich, Munich, Germany), 100 μg/mL ampicillin, 30 μg/mL kanamycin, and 10 % (v/v) exponentially growing culture of the reporter strain AI1-QQ.1. LB agar plates were coated with the top agar mixture. After 10 min, 5 μL of the plant extracts serially diluted in DMSO (25, 12.5, 6.25, 3.12, 1.5, 0.5, 0.25, 0.12 mg/mL) were applied, followed by a 1 h incubation at room temperature (RT) and overnight incubation at 37 °C. QQ activities were visualized by growth of the reporter strain [11].

### Data analysis

Data on antimicrobial activities and MIC were analyzed using SPSS software. All antimicrobial activities of plant extracts were compared with the standard antimicrobial agents. The mean inhibition capacity of plant extracts extracted using different solvents were compared using One-way ANOVA. Descriptive statistics were used to describe the Quorum Quenching activities of the candidate extracts.

## Results

### Screening for antimicrobial activity

The degree of inhibition, as determined by values of diameter of inhibition zone (IZ) of respective extracts, varied among the extracts with the highest inhibition being recorded for petroleum ether extract of seed of *Nigella sativa* against *Bacillus cereus* (IZ, 44 ± 0.31 mm), *B. cereus* ATCC 10987 (IZ, 40 ± 2.33 mm), oil from mature ripe fruit husk of *Aframomum corrorima* against *Candida albicans* ATCC 90028 (IZ, 35 ± 1.52 mm) and mature unripe fruit oil of *A. corrorima* against *S. aureus* (IZ, 25 ± 1.32 mm) (Table 2). The activities of oil from husk of *A. corrorima* against *C. albicans* and petroleum ether extract of seed of *N. sativa* against both laboratory isolated and reference strain of *B. cereus* were exceptionally high to the extent of exceeding the activity of control antibiotic (Gentamycin sulfate). Furthermore, oil extract from unripe fruit of *A. corrorima* had activity closer to that of Gentamycin sulfate (IZ, 28 mm) with inhibition zone diameter of 25 ± 1.32 mm against *S. aureus* DSM 346. *Pseudomonas aeruginosa* was the least responsive to any of the plant extracts from among the tested strains.

**Table 2:** Antimicrobial activities of selected plant extracts (500 mg/mL conc.) against test strains

Sample No.	Code	Inhibition zone diameter (mm) of plant extracts against reference strains					
		*E. coli* K12 DSM 498	*S. aureus* DSM 346	*B. cereus* ATCC 10987	*B. cereus* *Lab strain*	*P. aeruginosa* DSM 1117	*C. albicans* ATCC 90028
1	ACFA1	NA	10 ± 0.15	12 ± 1.5	23 ± 1.8	NA	15 ± 1.8
2	ACFA2	NA	NA	13 ± 1.08	15 ± 1.09	NA	12 ± 2.02
3	ACFA3	NA	NA	15 ± 1.32	13 ± 1.25	NA	10 ± 0.76
4	ACFO	20 ± 2.01	25 ± 1.32	NA	NA	12 ± 1.09	NA
5	ACHO	15 ± 1.71	NA	NA	NA	8 ± 0.88	35 ± 1.52
6	ASRM	12 ± 1.33	20 ± 1.04	15 ± 1.73	18 ± 1.82	NA	NA
7	CLRA1	NA	NA	10 ± 0.45	9 ± 1.73	NA	NA
8	CLRO1	NA	20 ± 1.89	NA	NA	NA	NA
9	CLRO2	15 ± 1.56	20 ± 0. 24	NA	NA	10 ± 1.09	NA
10	CLRA2	NA	NA	10 ± 0.59	12 ± 1.89	NA	8 ± 1.32
11	EBBP	NA	10 ± 1.33	NA	20 ± 1.22	NA	NA
12	JSSP	NA	15 ± 2.06	NA	20 ± 0.96	NA	9 ± 0.86
13	NSSO	10 ± 0.98	NA	NA	NA	8 ± 0.06	20 ± 2.18
14	NSSP	NA	NA	40 ± 2.33	44 ± 0.31	NA	16 ± 0.58
15	OSLC	NA	NA	12 ± 1.14	NA	NA	NA
16	VALC	NA	NA	22 ± 2.11	22 ± 1.78	NA	NA
17	VALM	NA	NA	14 ± 0.99	15 ± 2.11	NA	NA
18	VALP	NA	NA	NA	NA	NA	15 ± 1.24
19	Gentamycin Sulfate (1 μg/mL)	25 ± 0.012	28 ± 0.03	30 ± 1.18	25 ± 0.96	30 ± 1.05	ND
20	Nystatin (1 μg/mL)	ND	ND	ND	ND	ND	27 ± 2.33

Where: *NA* No activity, *ND* Not determined, Abbreviations of codes are as presented in Table 1

### MIC determination

The potency of candidate plant extracts were further evaluated after serial dilution of the original concentrated crude extracts to which the strains responded. Accordingly, MIC values of the extracts ranged between 12.5 to 25 mg/mL with the lowest MIC value (12.5 mg/mL) recorded for oil from husk of *Aframomum corrorima* against *P. aeruginosa* (Table 3). About 40 % (4/10) of the extracts with relatively better antimicrobial activities (closer to or even better than the control antibiotics) had MIC value of 25 mg/mL, while 50 % had MIC greater than 25 mg/mL.

**Table 3:** MIC of extracts of selected traditional medicinal plants of Ethiopia

	Concentration of extract (mg/mL)							
Extract/Test strain combinations	25	12.5	6.25	3.12	1.5	0.5	0.125	MIC (mg/mL)
ACFO / *S. aureus*DSM 346	+	+	+	+	+	+	+	>25
ACFO */E. coli* K12 DSM 498	+	+	+	+	+	+	+	> 25
ACFO /*P. aeruginosa* DSM 1117	-	+	+	+	+	+	+	25 l
ACHO/ *E. coli* K12 DSM 498	-	+	+	+	+	+	+	25
ACHO/*P. aeruginosa* DSM 1117	-	-	+	+	+	+	+	12.5 l
CLRO1 / *E. coli* K12 DSM 498	+	+	+	+	+	+	+	> 25
CLRO2 */E. coli* K12 DSM 498	+	+	+	+	+	+	+	> 25
CLRO2 */S. aureus* DSM 346	-	+	+	+	+	+	+	25
CLRO2/*P. aeruginosa* DSM 1117	-	+	+	+	+	+	+	25
DMSO + Nutrient broth	+	+	+	+	+	+	+	Control

Where: + = growth, - = No growth; for abbreviation of codes, refer to Table 1

### Quorum quenching activities of plant extracts

A total of 18 plant extracts were screened for Quorum Quenching activities using *E. coli* reporter strain AI1-QQ.1. Two of the plant extracts, namely Methanol extract of root of *Albiza schimperiana* (ASRM) and petroleum ether extract of seed of *Justica schimperiana* (JSSP), displayed observable QQ activities, suggesting the presence of non-toxic AHL interfering molecules in the extracts (Table 4). In addition to QQ activity, *Albiza schimperiana* had inhibitor activity against some test strains including *E. coli* K12 DSM 498 (Table 2, Table 4). Twelve of the extracts lack both inhibitory as well as QQ activity at concentrations assessed in the current study.

**Table 4:** Quorum Quenching activities of selected medicinal plants of Ethiopia

Extracts	AHL-QQ activity in *E. coli* based reporter strain AI1-QQ.1	Antimicrobial activity against *E. coli* K12 DSM 498
ACFA1	-	-
ACFA2	-	-
ACFA3	-	-
ACFO	-	+++
ACHO	-	+
ASRM	+	+
CLRA1	-	-
CLRA2	-	-
CLRO1	-	-
CLRO2	-	++
EBBP	-	-
JSSP	+	-
NSSO	-	+
NSSP	-	-
OSLC	-	-
VALC	-	-
VALM	-	-
VALP	-	-

Where, *AHL* Acyl homoserine lactone, *QQ* Quorum Quenching, *AI* Auto-inducer

+ = presence of QQ activity (as observed in reporter strain) or antimicrobial activity (as observed in standard reference strains); - = no activity; For abbreviation of codes, refer to Table 1

## Discussion

The emergence of drug resistant pathogens is making the treatment and control of infectious diseases more difficult. Among the common drug resistant pathogens are Multi-Drug Resistant TB (MDR TB), Methicillin-Resistant *Staphylococcus aureus* (MRSA), and Vancomycin-Resistant Enterococci (VRE). In 2013 alone, there were about 480 000 new cases of MDR-TB while extensively drug-resistant tuberculosis (XDR-TB) has been identified in 100 countries [31]. Because of poor performance, in accessibility, and high cost of the available drugs, traditional medicinal plants are getting public acceptance for the treatment of many infectious diseases.

Traditional medicinal plants have long history of application as alternative medicine. In the current study, extracts of different medicinal plants including leaf of *Vernonia amygdalina,* seed of *Nigella sativa,* and fruit of *Aframomum corrorima* displayed promising antimicrobial activity against medically important microbial strains with the highest inhibition zone diameter being recorded against *S. aureus* DSM 346 (IZ, 25 mm), *B. cereus* ATCC 11987 (IZ, 40 mm), laboratory isolated *B. cereus* (IZ, 23 mm), and *Candida albicans* (IZ 35 mm). Many of the observed antimicrobial activities were equal or even better than the activities recorded for control antibiotics. Our findings are in agreement with earlier reports on antimicrobial activities of extracts of *A. corrorima* [32], *N. sativa* [22], *Aframomum angustifolium* [33] and *Vernonia amygdalina* [34] where the antimicrobial activities were accounted, respectively, to the presence of phenolic compounds [32], phenol, tannin, saponnin and flavonoids [20], flavonoids and terpenoids [33], and combinations of some of the above phytochemical compounds including tannins, cardiac glycosides, saponnin and alkaloids [34].

Flavonoids are found in almost all parts of plant [35, 36] imparting color to flowers and fruits, and may protect the plant from insect pests and Ultraviolet radiations. Besides their antioxidant, anti-depressant and anti-inflammatory role in the human body, flavonoids act as bactericidal and bacteriostatic by damaging cytoplasmic membrane, inhibiting energy metabolism and synthesis of nucleic acids against different microorganisms [for review, refer to 35]. Flavonoids, including Quercetin, were reported to have antibacterial activity against *S. aureus*, *Bacillus subtilis*, *B. cereus*, *E. coli*, *Helicobacter pylori*, *Pseudomonas aeruginosa*, *P. fluorescens*, and *Enterobacter aerogenes* [35]. Flavonoid chalcone can be used as therapeutic agents against infections of Methicillin-resistant *S. aureus* strains [37]. The other common phytochemical isolated from traditional medicinal plants, is tannin. Tannins inhibit plasma coagulation by *S. aureus* [38]. It also form chelate with metal ions, and the possible antimicrobial mechanisms of tannins could be: induction of complexation with enzymes or substrates, act on the membranes of microorganisms; and toxicity due to complexation with metal ions [38].

In the current study, *Albizia schimperiana* displayed moderate activity against most of the test strains in agreement with it reported activities against bacterial (including methicillin-resistant *S. aureus and E. coli)* and fungal pathogens (*Aspergillus fumigatus)* [39]. The antimicrobial activities were assumed to be due to the presence of bioactive macrocyclic spermine alkaloid [39].

Although most of the evaluated plant extracts exhibited activities against the test strains, the lowest MIC recorded for fruit husk oil of *A. corrorima* against *P. aeruginosa* (12.5 mg/mL) was relatively lower than the MIC reported for ethanolic extract of *V. amygdalina* (25 mg/mL) against *P. aeruginosa* [38] but higher than the < 4 mg/mL value recorded for 36 medicinal plant species evaluated in Peru [40]. The overall MIC values recorded in the present study fall within the wider MIC range (0.008 to 256 mg/mL) reported for many of the 141 medical plants of Peru [40] although none displayed strong activity with MIC value < 12.5 mg/mL.

In addition to antimicrobial activities, extracts from traditional medicinal plants could interfere with bacterial cell-cell communication. In the current study, two of the eighteen plant extracts (11 %) were found interfering with bacterial QS. Likewise, Allison et al. [41] reported 12 % (6/50) isolation rate of medicinal plant that exhibit anti-Quorum Sensing activities as evaluated using the two biosensor strains, *Chromobacterium violaceum* and *Agrobacterium tumefaciens.* In a complex ecosystem where plant, soil microorganisms and abiotic factors are in a constant interaction, constitutive production of signaling molecules among bacterial population and anti-Quorum Sensing molecules by a plant against potentially pathogenic microbes is a natural phenomenon [42]. The behavior of a natural multi-species community is likely to depend at least in part on co-existing QS and Quorum Quenching (QQ) activities [42]. Like all other plants, medicinal plants could also produce signaling molecules and possibly rely on QS and Quorum Quenching (QQ) activities while interacting with the biotic components the ecosystem making them candidate sources of QQ substances in the fight against microbial pathogens.

Generally, biological compounds usually target the bacterial AHL-QS system via three different ways: stops the signaling molecules from being synthesized by the *luxI* encoded AHL synthase, degrades or modify the signaling molecules and/or target the LuxR signal receptor [9, 43]. In our case, the plant extracts most likely interfered with AHL signal molecules either through degradation or modification by proteins, or by antagonizing activities of small molecule in the extract (such as 3-oxo-C6-AHL). For wise use of the current finding, the detailed mechanism of action(s) of Quorum Quenching substance(s) need to be determined (one of the limitations of this study). While bacteria use Quorum Sensing communication circuits to regulate a diverse array of physiological activities [44–47], Quorum Quenching prevents the correct operation of Quorum Sensing. The finding that plants have developed Quorum Quenching mechanisms indicates that the plant developed strategies to protect itself against pathogens. Accordingly, the Quorum Quenching activities observed in the current study could mainly be accounted to the presence of auto-inducers (AHL) inhibitors in our plant extracts.

Based on the current findings and many of the earlier observations, traditional medicinal plants will continue to be the source of alternative antimicrobial substances to combat the challenges being confronted due to emergence of drug resistant microbes. Series of reports confirmed the high prevalence of drug resistant bacterial strains in both clinical and food samples [22, 48–50]. Increased prevalence of drug resistant bacteria, together with lack and/or high costs of new generation drugs have been escalating infection-related morbidity and mortality particularly in developing countries like Ethiopia [24]. Thus, detection of plants with potent activity against pathogenic microorganisms in the current study as supported by earlier reports from Ethiopia [17, 19–22] further strengthen the feasibility of looking for an alternative approach to manage drug resistant microbes. As stressed by many scholars, using effective plant extracts to control human diseases has the additional advantage of low production costs, minimal environmental damage and higher accessibility to rural communities [23, 51].

## Conclusion

Based on the current finding and the available literature, it could be concluded that medicinal plants have significant role in human medication, displaying both antimicrobial and anti-Quorum Sensing activities. The latter case is safer as it will not kill bacteria and the likely chance of development of resistance is low, if any. The fact that the petroleum ether extract of *N. sativa* displayed even better activity against *B. cereus* strains as compared to the commercial control antibiotic, Gentamycin sulphate, reveals the future prospects of traditional medicinal plants of Ethiopia in the treatment of many diseases and will be source of many biochemical molecules of therapeutic importance.

Furthermore, the anti-QS activities observed among two of the Ethiopian traditional medicinal plants is good indication of potential application of plant extracts for the regulation of microbial physiology in the way it fits human interest. Thus, Quorum Quenching could be an alternative strategy to combat bacterial infections as it lowers the development of multidrug resistant pathogens. In addition, as plants are constantly exposed to bacterial infections, similar to humans and other animals, it is logical to expect that plants have developed sophisticated chemical mechanisms to inhibit biofilm formation and other microbial pathogenesis. Being the first assessment of its kind on the potential application of Ethiopian traditional medicinal plants for interference in microbial cell-cell communication (anti-Quorum Sensing activities), the detailed chemistry of the active compounds and possible mechanism(s) of actions of the bio-molecules responsible for the observed interference were not addressed in the current study. Thus, further evaluation for the nature of those active compounds (bio-molecules) and detailed mechanism(s) of their interaction with microbial processes are recommended

## Abbreviations

AHL, acyl homoserine lactone; AI, auto-inducer; ATCC, American type culture collection; DMSO, dimethyl sulfoxide; DSM, Deutsche sammlung von mikroorganismen (German Collection of Microorganisms and Cell Cultures); IZ, inhibition zone; MDR TB, multi-drug resistant TB; MHA, Muller Hinton Agar; MIC, minimum inhibitory concentration; MRSA, methicillin-resistant *Staphylococcus aureus*; PDA, potato dextrose agar; QQ, quorum quenching; QS, quorum sensing; VRE, vancomycin resistant enterococci; XDR-TB, extensively drug-resistant TB
